# General Treatment of Fractures, a Clinical Lecture*A clinical lecture delivered in Bellevue Hospital and stenographically reported by E. Dolan for the Southern Medical Record.

**Published:** 1893-02

**Authors:** F. S. Dennis

**Affiliations:** New York City, Professor of Surgery in Bellevue Hospital Medical College


### Southern Medical Record.

A MONTHLY JOURNAL OF MEDICINE AND SURGERY.

VOL. XXIII. ATLANTA, GA., FEBRUARY, 1893. No. 2.

### Original Articles.

### GENERAL TREATMENT OF FRACTURES.*

BY F. S. DENNIS, M. D., NEW YORK CITY,
Professor of Surgery in Bellevue Hospital Medical College.

GENTLEMEN :—I present to-day a large number of patients suffering from various kinds of fractures in different parts of the body. Before taking up the special treatment of each case, I wish to make some remarks applicable to fractures in general. We will not consider here the symptoms of fracture; we'll assume that the bone is broken and that we are going to treat it in the proper way.

In the first place, a fracture should receive your immediate attention. You can't neglect it for a day or two, for in that time changes may take place, the results of which will be impossible for you to remedy.

It is important to move a fractured limb in the proper way. Never grasp it at the point of injury; for instance, notice the manner in which I move the limb of this patient, who has sustained a fracture of the lower half of the leg. I take hold of the foot near the toes and make strong traction in the long axis of the limb. This overcomes any involuntary contraction

*A clinical lecture delivered in Bellevue Hospital and stenographically reported by E. Dolan for the Southern Medical Record.

of the muscles, which the pain caused by movement of the part sets up, and thus prevents a simple from being converted into a compound fracture. The more he resists you, the stronger must be your extension. This is not only less painful and dangerous than getting under the fractured part and lifting it up, but you notice that it is a relief to the already existing pain. This same rule applies to fractures in any part of the lower or upper extremity.

When you see a person who has a fracture, keep him in the recumbent position. Carry him to his home or to a hospital on a stretcher; allowing him to sit up in a carriage is very apt to convert a simple fracture into a compound. Never keep such a patient on a feather bed or on a soft bed of any kind, but put him on a hard mattress.

The plaster of Paris splint is the best for general use. Some surgeons object to it, saying that it causes edema, pain, inflammation, etc. As a matter of fact, these are usually the result of an improperly fitting splint; the plaster splint should be applied immediately upon the receipt of the fracture, when it will prevent these symptoms. There are some contraindications to its immediate use, as when there are blebs, sloughing or great tension over the part, or an extravasation of blood around the seat of injury. When these conditions exist, remove them and then apply the plaster dressing; make an incision or put in a trocar and allow the blood to escape; snip off a bleb with a pair of scissors and apply iodoform; wait until tension and sloughs subside, if they be present, then apply the plaster dressing. If muscular contractions prevent the proper adjustment of the fragments, do not hesitate to do tenotony, even if you have to cut the tendo-Achilles; it can do no harm, for your patient will be in bed several weeks as a result of the fracture, and a divided tendon unites in two weeks, so it is all right long before he wants to use it. This muscular contraction may be the cause of a compound fracture resulting from a simple one. You can also place the limb in such a position as to relax the muscles and thus overcome muscular irritability; as you see we do in this man with a Potts' fracture of the leg; I flex the leg on the thigh and the foot on the leg; this muscular action is a temporary trouble, lasting only while the limb is being set; where you don't want to do a tenotony, give large doses of morphia or sulphonal for this muscular action.

As I before remarked, apply a plaster of Paris splint in the vast majority of cases. A serious objection to it is the danger of getting it on too tight. Gangrene has resulted several times from this mistake. Remember the points where the arteries lie superficial and where compression would stop their flow, as at the ankle. Pressure can do no harm in the middle of the leg where the arteries lie far down in the tissues. If you fear pressure from subsequent swelling, place layers of borated cotton next to the skin; this equalizes pressure as the limb increases in size. There is no danger of gangrene or slough where cotton is used in this way. If, on the other hand, the limb gets too small for the dressing, cut out a longitudinal strip and bring the splint together with an extra bandage or two.

In a fracture of the leg, prevent backward displacement. Do this by putting a perforated tin splint on the posterior part of the leg, between the layers of the plaster dressing. The perforations in this tin makes each side of it rough and easily caught up by the dressing, thus it can't bend or move. This greatly increases the strength of the dressing without adding materially to its weight; the same is applicable to the sides of the limb. Use strips of tin about an inch wide and eighteen inches long. These often prevent shortening of a fractured limb.

A fracture should be looked at every week. I have treated over a thousand compound fractures and five times as many simple ones and I have found from experience that every fracture, no matter whether you think it is all right or not, should be examined every week. If you find it is not all right at the end of the first week, it is not too late to reset it, but if you neglect it for a couple of weeks, the deformity that results, if it had been improperly set, can only be remedied by an osteotomy. You need not remove the whole dressing to do this; simply cut a window over the seat of injury.

If the patient complains of pain after your dressing is applied, remove the dressing or cut out a window and find the cause, for if it's properly adjusted, there should be no pain. This is nature's method of informing you that something is wrong and if you neglect to attend to it, the lawyer will have a good point against you in the law-suit that is apt to follow. Pain may be caused by too much compression at a point, an improper adjustment of the fragments, etc., and should be relieved at once by removing the cause.

We have here a patient with a fracture of the patella. Wiring is the proper treatment if your patient is in proper condition; this kind of fracture is an exception to the rule that permanent dressings should be applied immediately, for there is usually so much effusion in the joint that a dressing applied early would soon be too large; so we must wait until the effusion is absorbed.

This next patient has a fracture in the middle of the femur, His habits are those of a chronic alcoholic and you see that he has a mild form of delirium tremens. This is a very frequent result of a fracture in an habitual drunkard. Such a patient is hard to treat; he'll get up and walk around the ward with a broken leg. We'll place this limb in a plaster splint, tie the patient down and give large doses of chloral and bromides and some alcohol. We had two other cases of patients with delirium tremens the result of fracture of the leg, but both died yesterday. This is a serious condition.

This next man has a simple fracture of the humerus. We get a false point of motion, crepitance, loss of function, etc. We move his arm by making strong extension on his hand, at the same time telling him to relax his muscles or let himself "go loose." The musculo-spinal nerve, which runs in a groove at the posterior part of the humerus, is apt to be involved here by injury at the time of the accident or by callous subsequently thrown out. Inform your patient early that impairment of the function of the arm may result from this involvement, and thus partially protect yourself. We'll put a plaster splint on him immediately.

We have here a couple of patients with Colles' fracture of the lower part of the arm. Such fractures usually occur from patients falling on their hands. Every slippery day in New York City, Bellevue Hospital receives several of them. The plaster splint will be applied to both.

We have a couple of patients here with compound fracture of the leg. Formerly such accidents were fatal. They nearly all died of pyemia unless amputation was performed, which in itself did but little to save the patient's life. Sixty-eight per cent. used to be the mortality in compound fractures. In the days of antisepsis and modern surgery it is one-half of one per cent. I haven't seen a case of pyemia in this hospital in the last ten years as the result of an operation or a fracture. The same general rules apply here as in simple fracture. Get the limb in plaster of Paris as soon as possible. Must be careful about sepsis. Wash the whole limb, foot, toes and all, in soapsuds ; irrigate with clear water, then shave and pour over it a solution of 1 to 500 bichloride. Then when the whole limb is clean, attend to the wound. Open it thoroughly, put the nozzle of a syringe in the bottom of it and wash out with 1 to 2,000 bichloride. Then with forceps and scissors cut away and remove all loose shreds of muscle, fascia or any other tissue. Remove particles of clothing, machine oil or foreign bodies of any kind. Then make an incision through the wound, out the under surface of the limb, in order to provide good drainage for the outflow of serum caused by the antiseptic solutions. Close up the anterior wound entirely and leave the drainage tube in the posterior wound for three days. Remove the tube and examine the part by making a window in the plaster dressing which should be applied immediately after the injury. That was the treatment in this patient who had a compound fracture of the femur, and you see that not a drop of pus has formed. It is a perfect result of antiseptic surgery. We can dress the wound through the window in the dressing and can move him about with perfect safety. When you see gauze or cotton saturated with the bloody serum around a fracture, remove it, for when in this condition it is a good hot-bed for the development of germs.

In a compound comminuted fracture, remove all particles of bone not covered with periosteum ; if they are covered, leave them there for they will be nourished and you will need all the fragments possible to render the union firm.

We notice on this patient's leg a small bleb ; before applying the dressing we'll open it and allow its contents to run out, then cover with an artificial scab made out of iodoform and collodion, thus avoiding the probability of the bandage converting the bleb into a slough.

To sum up, use plaster of Paris dressing for splints in nearly all cases, apply it immediately, look at a fracture every week, lift a limb by making extension at its extremity, and prevent backward displacement by applying narrow strips of perforated tin between the layers of plaster posteriorly.

